# Teacher agency in the times of crisis: a situational analysis of school environment after the 2022 Russian invasion in Ukraine

**DOI:** 10.3389/fpsyg.2024.1382403

**Published:** 2024-05-23

**Authors:** Anna Szczepaniak-Kozak, Emilia Wąsikiewicz-Firlej

**Affiliations:** ^1^Institute of Applied Linguistics, Adam Mickiewicz University, Poznań, Poland; ^2^Institute of East Slavic Studies, Adam Mickiewicz University, Poznań, Poland

**Keywords:** teacher agency, teacher empowerment, emerging multilingualism in Polish schools, crisis situation, Ukrainian refugee pupils, family-school cooperation

## Abstract

From February to May 2022, the war in Ukraine prompted Poland to accommodate 3.37 million refugees from conflict zones, in addition to 850 thousand Ukrainian economic migrants already residing in the country. A substantial proportion of these refugees, primarily mothers with children, swiftly integrated into the Polish educational system, with some children commencing schooling within a week of their arrival. This influx significantly diversified the then predominantly monolingual landscape of Polish schools. Given the uniqueness of this situation and the fact that Poland has historically remained mono-national and monolingual for decades, Polish teachers suffered from a lack of preparedness, resources and expertise to effectively navigate their teaching practices in multilingual classes. To understand the specificity of this situation, taking especially into account the perspective of educators, we have designed a qualitative study drawing on focus group and individual interview reports. We were particularly interested in determining how teachers' agency was activated in times of crisis. The findings reveal how the newly-emerging linguistic and cultural heterogeneity is perceived by teachers, how it is manifested in school and home environments, and the extent to which possibilities for synergies exist between the two. The findings also highlight the fact that, despite teachers' inexperience and unpreparedness for the new educational context, they instantly responded to the challenges that emerged. This can be exemplified by teachers' collaboration in material design as well as the willingness to participate in courses sensitizing to migrant students' needs (e.g., linguistic, educational, or emotional ones).

## 1 Introduction: rationale, gap in research, research questions, structure

Between February and December 2022, due to the war in Ukraine, Poland admitted 3.37 million refugees from conflict zones, who were added to 850 thousand Ukrainian economic migrants already living on its territory. As reported by UNHCR (2023), in mid-2023, over one-quarter of the Ukrainian population continued to be displaced, and around 1 million Ukrainian refugees still resided in Poland. Most refugees are mothers with children, some of whom started schooling in Poland within about a week of their arrival. To show the scale of the increase in multiethnicity of Polish schools, in 2009, 9,610 non-Polish pupils were schooled in Poland and in 2019, the figure was 51,363. However, just between January and May 2022, the number of Ukrainian students in the largest cities rose by over 106% (Unia Metropolii Polskich, 2022). According to the most recent statistics, in January 2023, 190,000 Ukrainian learners (including kindergarten children) continued their education in Poland in 20,557 educational institutions (Otwarte Dane, 2023), which is 5% of the general pupil population. Due to the unprecedented nature of these circumstances, and the fact that Poland for decades was almost a monoethnic country, Polish teachers lacked the knowledge, tools, and expertise to deal with the numerous challenges which are characteristic of multilingual classes.

In light of Bourdieu's (1998) ideas, newcomers bring their *linguistic capital* to *the linguistic market*, which was exactly the case with Ukrainian pupils, who enriched the school linguistic environment with their first languages, i.e., Ukrainian or/and Russian. Bourdieu (1998) proposes that the possession of linguistic capital may transform into educational, economic, cultural, demographic and general social capital. These capitals mutually reinforce one another and are necessary for maximizing the potential of particular individuals and groups in society. Furthermore, migration always establishes the value of individual language resources that need to be renegotiated, since migrants might not be able to use their languages in the work or schooling environment, prioritizing or privileging society-dominant languages. In consequence, it might result in language loss among first and second-generation migrants which is considered a common trend worldwide (Capstick, 2020, p. 17).

The emergent multilingual turn in Polish schools has, unfortunately, rarely been seen as an asset by school personnel, teachers included, routinely dealing with monolingual classes and unprepared to work with multilingual pupils. In the first weeks following the Russian invasion and the influx of Ukrainian pupils to Polish schools, the main concerns included overcoming the communication barriers and settling the children into the new educational system. Most of the school staff's efforts were thus focused on the pupils' integration, curriculum and learning of the Polish language, rather than their language resources. Later on, instead of capitalizing on newly-arrived pupils' multilingual repertoires, teachers perceived this diversity as a challenge, not to say an obstacle, hindering their teaching practices.

In our paper, we posit that teachers play a particularly significant role in the lives of migrant pupils and their families and, in the long run, they might have a tangible impact on the pupils' linguistic capital, which can influence their future educational and career paths (Kim and Kim, 2016). However, until recently, little notice was paid to how the admission of larger groups of pupils with migration backgrounds affected schools, specifically on the role and response of teachers in this context. We also aim to bridge the gap in research on teachers' support, or lack of it, for the maintenance of pupils' linguistic capital (Sook Lee and Oxelson, 2006, p. 456; Szczepaniak-Kozak et al., 2023). Our inquiry is specifically aimed at determining how teachers' agency was activated in times of crisis, and identifying the determinants that influence their actions and decisions in these circumstances.

To encapsulate our research aims, we have formulated the following research questions (RQs) to guide our investigation:

RQ1: What elements of the situation in which Polish teachers found themselves in 2022 influenced their agency?

RQ2. How did teacher agency manifest in the crisis situation triggered by Russia's invasion of Ukraine in 2022?

RQ3: How is the newly-emerging linguistic and cultural heterogeneity perceived by teachers once the school situation stabilized, i.e., at least 9 months after the invasion?

The theoretical framework for the study draws on Priestley et al.'s (2016) Ecological Model of Teacher Agency and Bronfenbrenner's (1979; 1992; 2005) Ecological Systems Theory, both of which will be outlined in the following sections. Theoretical considerations are then followed by the research report on the study undertaken and the discussion of our findings, with outlets for its impact.

## 2 Theoretical framework: home language(s) loss and the role of teacher support

### 2.1 Home language(s) loss and the role of schools in preventing it

As mentioned earlier, first language loss in first and second-generation migrants appears to be a common phenomenon. A rapid decline in fluency in the first language in the early years of schooling occurs when minority or home languages are not fostered within the school environment (Cummins, 2005, p. 586; Szczepaniak-Kozak et al., 2023, p. 118, 119). Even at the preschool stage, young children discern the difference in status between their home languages, usually holding a minority language status in society, and the majority language. In Bourdieusian terms, children quickly recognize the value of particular languages in the linguistic market and use those that hold a more powerful position in society. Since the language of schooling tends to be society's dominant official language, it is typically chosen by children. Additionally, when educational interactions with teachers substantiate and perpetuate these distinctions, adolescents may become detached from their minority identities or home languages, hastening the progression of language loss (Cummins, 2005).

Scholars point to a number of sociolinguistic factors fostering home language (HL) maintenance, including child agency (e.g., Schwartz and Mazareeb, 2023), parental support, interaction with siblings and relatives, HL-speaking community and other social networks, as well as the school environment (family-school partnerships). Taking into account the significance and importance of all these determinants, HL supportive educational environment and formal HL instruction seem to play a detrimental role in HL maintenance (see e.g., Banasiak and Olpińska-Szkiełko, 2020; Szczepaniak-Kozak et al., 2023, p. 134–144). Teachers' positive attitudes and respect for pupils' HL appear to play a crucial role in students' inclination to preserve their HL (Ball and Lardner, 1997; Corson, 2001; Nieto, 2002; Macías, 2004). When linguistic minority pupils perceive that their HL or cultural background is deemed inappropriate or undervalued in the school settings, they are prone to disassociate from their HL and abandon it (Lanehart, 1998; Wong Fillmore, 2004).

Despite their significant role in HL maintenance, teachers, especially those not exposed to relevant language-sensitive training, tend to manifest negative or indifferent attitudes toward HL maintenance and multilingualism and do not seem to understand the critical role of HLs in the personal, academic and social development of minority pupils (Sook Lee and Oxelson, 2006; Szczepaniak-Kozak et al., 2023, p. 21–52). Importantly, fostering pupils' HL maintenance does not require teachers' proficiency in pupils' HL (Sook Lee and Oxelson, 2006). It is often enough to express interest in HL and perceive it as a resource for teachers to reinforce their pupils' drive to maintain their HLs (Franquiz and de la Luz Reyes, 1998).

The existing research findings highlight a noteworthy correlation between teacher attitudes, beliefs, and their actual teaching practices. The data indicate that unless teachers truly value the advantages of multilingualism and comprehend the detrimental impact of losing one's home language, it is improbable that the needs of HL speakers will capture teachers' attention or align with their interests. For example, Sook Lee and Oxelson (2006, p. 468) emphasize the pivotal role of teachers in acknowledging the significance of HLs for pupils from linguistic minority backgrounds. The scholars underscore that such recognition is crucial for fostering the holistic development and empowerment of these students. They further emphasize the need for educators to prioritize HL maintenance, making it more visible on educational agendas and teacher training curricula [see good practices in Gogolin et al. (2011); Little and Kirwan (2021); Szczepaniak-Kozak et al. (2023), p. 203–232].

Wong Fillmore (2004, p. 339) asserts that the future of multilingual education and addressing the challenges faced by minority students hinges on the “willingness of educators and everyday individuals to embrace linguistic and ethnic diversity, particularly within our educational institutions.” Furthermore, in her work (ibidem), the scholar highlights several factors that prompt teachers to reflect professionally, including how to address the language needs of students who are not proficient in the language of instruction, approaches toward supporting families' and communities' efforts to preserve their heritage or home languages, and the accommodations schools should provide for students who are not proficient in the language of instruction. However, schools continue to be recognized primarily as the catalyst in helping migrant children acquire proficiency in the majority language. Even though Wong Fillmore made this observation about U.S. schools in 2004, two decades later it remains relevant to schools in Poland, where teachers are seen as responsible for enabling pupils to become proficient in the majority language (Polish), often without seeking forms of accommodating differentiated needs and learning in the linguistically and culturally diverse classroom (Szczepaniak-Kozak et al., 2023, p. 51), taking into account input from the affected communities.

Given that large-scale migration is a relatively recent phenomenon in Poland, the paper's focus is not on investigating societal conditions or systemic changes enabling pupils with migration backgrounds to receive multilingual education, but on what teachers can do daily to provide them with meaningful and comprehensive opportunities to engage in the educational program offered, tapping into the potential that their entire linguistic capitals enable. This perspective aligns with research findings indicating that when pupils abandon their first language to assimilate quickly into a new environment, they risk “losing their native languages and struggling to communicate with their own families and communities” (Wong Fillmore, 2004, p. 349). While multilingual education may not entirely prevent language and cultural erosion, it can sufficiently slow down the process, facilitating a smoother adjustment for young migrants and their families in new environments (ibidem).

In this context, teachers' competencies and mindsets play a significant role in creating a supportive environment for HL maintenance. For instance, Daase et al. (2023, p. 54) advance the notion of *contingency competence* as a pivotal factor in enhancing educational opportunities for children with migration experiences, which is defined as “the sensitivity and awareness of the principal openness of human life forms and their diverse possibilities for linguistic, material, and practical expression.” This competence, as delineated, extends beyond the context of newcomers within specific communities of practice, such as schools or classes, to encompass the entire school ecosystem and its stakeholders. Instead of concentrating solely on established standards or patterns of behavior, the focus turns to acknowledging the innate “openness and non-essential nature of human lifeforms,” largely influenced by language and society (ibidem: 71). Thus, at the core of this notion, lies the significance of being attuned to and perceptive of numerous modes of both material and linguistic expression that come to the fore in multilingual school environments. Encouraging pupils to tap into their entire linguistic capital helps to foster their agency and supports societal inclusion in the longer run.

Recognizing the pivotal role teachers play in creating a supportive environment for pupils' HL maintenance, the following section will delve into the concept of teacher agency within the framework of an ecological perspective.

### 2.2 Teacher agency from an ecological perspective

Agency stands out as one of the most ambiguous and contentious terms in the realm of education. It has been associated with several notions, including e.g., “selfhood, motivation, will, purposiveness, intentionality, choice, initiative, freedom and creativity” (Emirbayer and Mische, 1998, p. 962). Another commonly construed meaning of agency revolves around the notion of action, often framed in opposition to certain social structures. A logical consequence of conceptualizing agency in this manner is its interpretation as an *innate capacity* of the human species. In this sense, particular individuals may possess innate levels of agency that differ from one another. Alternatively, agency can be understood as an emergent phenomenon, cultivated by individuals through the dynamic interaction of innate capacities, and the varied array of resources, opportunities and constraints existing in the environment where the individuals are situated. This conceptualization could be deemed ecological, as it integrates the influence of both individual capacity and contextual variables in shaping agency, while underscoring its temporal dimension (Priestley et al., 2016, p. 20). Bearing in mind the complexity of individual and external variables that shape teachers' work, in this paper, we adapt Priestley et al.'s (2016) ecological approach to agency. In his proposition, agency goes beyond individuals' capacities and engagements, taking into account temporarily restricted situational and societal variables shaping their actions.

In educational settings, teacher agency tends to be construed as professionalism, accountability or educational change, frequently championed as a slogan endorsing educational policies (Priestley et al., 2016, p. 26). Oolbekkink-Marchand et al. (2017, p. 38) believe that individuals go beyond reacting and replicating established practices. Instead, they demonstrate the capability to take independent action, deliberately creating and improving their surroundings to assert control over their lives. The term “teacher agency” refers to teachers' capability to take intentional, meaningful action that manifests their will, autonomy, independence and choice. Within the professional domain, agency signifies a teacher's ability to transcend contextual rules and regulations, allowing them to pursue their own objectives (ibidem).

Certain conceptualizations of agency align with the ecological perspective, offering avenues to articulate and understand it as a construct. For instance, Lasky's (2005) sociocultural conceptualization of teacher agency underscores its dual nature, dependent both on the individual and situational factors, intricately interwoven with “culturally, socially and historically developed” resources (ibidem: 900). It may include teachers' contingency competence, seen as situated school/classroom performance in reaction to a real-life cluster of factors. In a similar vein, Pyhältö et al. (2012, 2014) advocate for the feasibility of teachers exercising agency through their relational and temporal connections, i.e., via a network of “interactions between teachers, pupils and their parents, as well as with other members of the school community” (Pyhältö et al., 2014, p. 337). These conceptualizations are encapsulated in the ecological model of teacher agency (Priestley et al., 2016) which informs our research.

The model comprises three dimensions: iterational, projective, and practical-evaluative. The iterational dimension acknowledges the influence of teachers' past experiences and capacities, encompassing both personal and professional realms. The practical-evaluative dimension discerns cultural, material, and structural facets, while the projective dimension delineates between short- and long-term orientations of teacher agency.

As far as practical-evaluative aspects of teacher agency are concerned, cultural aspects capture patterns of thinking and speaking as well as the systems of values, beliefs and aspirations, articulated in the internal and external dialogues. Material aspects represent the affordances available or unavailable in a given physical setting, influencing the facilitation or hindrance of teachers' actions. Structural aspects pertain to social structures and networks that impact agency. This model emphasizes individual and situational aspects of agency that can be enacted in a specific, temporal context. It is molded by the amalgamation of past experiences, including formal education and informal personal and professional experiences, future orientations guided by personal ambitions and values, as well as all tangible and intangible resources available in a given situation (Priestley et al., 2016, p. 30).

We find the application of this approach particularly relevant in the context of teacher agency in a crisis situation – specifically, the emergent influx of war refugees into the Polish school system, as well as the contingent transition from a monolingual to a multilingual school environment.

This approach further resonates with Bronfenbrenner's (1979, 1992) Ecological Systems Theory of Human Development and its revised version (2005). It advances the idea that a developing individual is impacted by the complex network of interactions with and within their immediate environment over time, conceptualized as embedded structures (sub-systems) at **five** different levels, i.e., the microsystem, mesosystem, exosystem, macrosystem and chronosystem. Over time, Bronfenbrenner (2005) also recognized the relevance of the biological and genetic characteristics of the individual.

The microsystem signifies the immediate environment of the individual, which embraces the activities, roles, interpersonal relations and lived experiences of a person situated in a given physical and material setting “where people can readily engage in face-to face-interaction” (Bronfenbrenner, 1979, p. 22). Bronfenbrenner (1979) also emphasized the salience of individual traits and experience and those aspects of an environment that give meanings to individuals. Importantly, the perception of a given situation or environment relies not only on its objective characteristics but also on subjective interpretations. This micro level (as well as the personal level) corresponds with the iterative dimension of agency (Priestley et al., 2016), encompassing the teacher's individual characteristics and personal traits that shape agency, such as personal experiences, backgrounds, values, beliefs, and emotions.

The mesosystem stands for interrelations between two or more systems in which an individual participates (Bronfenbrenner, 1979). In other words, these interrelations might be interpreted as a connection between different settings. In the school environment, the meso level corresponds with the local, institutional level, involving interactions between teachers and pupils, colleagues, school management and neighboring schools. The mesosystem interplays with all dimensions of teacher agency, i.e., the iteration, practical-evaluative and projective ones.

Bronfenbrenner (1979, p. 237) also delineates the exosystem as “one or more settings that do not involve the developing person as an active participant but in which events occur that affect, or are affected by, what happens in that setting.” While certain events may not directly involve an individual, their impact on that person persists. This impact might also manifest in the reverse direction. For instance, in the case of teachers, the exosystem might stand for the pupils' home environment, especially parents. Even though teachers are not inherently integrated into this particular setting, their influence upon it and the reciprocal impact from it are evident.

The macrosystem, as construed by Bronfenbrenner (1979, p. 258), encapsulates the consistency prevalent within a culture or subculture across its microsystem, mesosystem, and exosystem, alongside any underlying belief systems, ideologies or even narrative frames. This overarching perspective broadly mirrors the encompassing cultural milieu within a given environment. In the educational domain of teaching practice, the macrosystem extends to the broadest national level of teachers' work. This includes collaboration with colleagues across diverse schools and organizations at regional and national levels, involving various stakeholders. Furthermore, it considers the influence of top-down policies and legislative frameworks that govern the national educational systems, along with the available resources that significantly impact educators and their work environments.

The chronosystem was later introduced into Brofenbrenner's original theory (Bronfenbrenner, 1992) to encapsulate the dynamic nature of any given environment that undergoes changes over time. These changes exert influence across all systems, including life changes at an individual level. Accordingly, as individuals progress through developmental stages, their learning patterns and interactions with ecological systems continually evolve, thereby shaping their cognitive, social and emotional growth.

Our further analysis and interpretation of research findings will attempt to unveil the multifaceted nature of teacher agency and its situatedness in a complex mosaic of individual, cultural, and societal factors that might be compared to “nested Russian dolls to describe the layers of relationships” (Leonard, 2011, p. 1004).

## 3 Methods

To understand the specificity of Polish teachers' agency profile, given the uniqueness of Poland's socio-educational situation, especially the fact that it remained mono-national and monolingual for decades, we have designed a qualitative study drawing on focus groups and individual interview reports. We were particularly interested in determining how teachers' agency was activated in times of crisis.

### 3.1 Research design (including the sample)

This paper constitutes an initial exploratory investigation into an unprecedented situation. To collect valid and reliable data, we began by conducting a case study. This involved visiting a primary school that admitted a relatively large group of Ukrainian refugee pupils on an emergency basis after the war broke out. In this pilot research, we were able to take a first glimpse into the needs and first reactions of teachers and also to conduct pilot interviews with school personnel. Our interview questions were organized, in line with Bronfenbrenner (1979; 1992; 2005) Ecological Systems Theory of Human Development, into five modules: those asking about the teachers' microsystem, mesosystem, exosystem, macrosystem and chronosystem, in order to indicate how these shaped their agency. After the piloting stage, we were able to adjust the interview script and choose the most adequate data elicitation method: Individual and Focus Group Interviews, fortified with real and virtual artifacts, for example, those from schools' websites and classroom materials prepared by teachers, in line with the Situational Analysis assumption that the data coming from qualitative interviews “ideally will include all kinds of extant discourse materials found in the situation of inquiry broadly conceived” (here schools) (Clarke et al., 2022, p. 9). The reason why some interviews were conducted in a group format was that, due to problems with gathering robust enough data, in some cases, we needed to resort to convenience sampling. That is to say, some of the interviews took place after teacher training which the authors of this paper conducted, and instead of interviewing individual teachers, entire groups were invited to provide their feedback to the questions included in the interview script (Appendix 1).

Altogether, we surveyed 37 school staff members and other professionals involved in education and teacher training. The participants in our interviews were as in Table 1, where FG stands for a focus group accompanied by its number, IR stands for an individual interview accompanied by its number, n stands for the number of participants in a particular research session (Table 1).

**Table 1:** Respondents' profile (type of school they work for and years of experience).

**Respondents' data**		
**Focus groups (FG)**		**Individual respondents (IR)**
FG1: *n =* 5	R1: a primary school teacher; experience: 5 years	IR: a school principal; primary school; experience: 20 years
	R2: a primary school teacher; experience: 8 years	IR2: a teacher of Polish; primary school; experience: 10 years
	R3: a primary school teacher; experience: 4 years	IR3: a teacher of Russian; primary school: experience: 1 year
	R4: a primary school teacher; experience: 12 years	IR4: a teacher of English and teaching assistant for children with disabilities; secondary school; experience: 19 years
	R5: a secondary school teacher; experience: 16 years	IR5: an early school education teacher; primary school; experience: 3 years
		IR6: a kindergarten teacher; experience: 5 years
		IR7: a biology teacher; primary school: experience: 34 years
		IR8: a teacher of Polish; vocational secondary school: experience: 50 years (retired, working part-time)
FG2: *n =* 7	R1: a primary and secondary school principal; experience: 20 years	IR9: a teacher of Polish; vocational secondary school; experience: 24 years
	R2: a cultural assistant; experience: 12 years	IR10: an early education teacher and librarian; primary school; experience: 7 years
	R3: a primary school teacher; experience: 11 years	
	R4: a primary/secondary school teacher; experience: 10 years)	
	R5: a primary school teacher; experience: 4 years	
	R6: a primary school teacher; experience: 8 years)	
	R7: a primary school teacher; experience: 2 years	
FG3: *n =* 15	R1: a cultural assistant; experience: 2 years	
	R2: a cultural assistant; experience: 2 years	
	R3: a cultural assistant; experience: 1 year	
	R4: a cultural assistant; experience: 1 year	
	R5: a cultural assistant; experience: 1 year)	
	R6: a cultural assistant; experience: 1 year)	
	R7: a cultural assistant; experience: 1 year)	
	R8: a cultural assistant; experience: 1 year	
	R9: a cultural assistant; experience: 1 year	
	R10: a primary school teacher/cultural assistant; experience: 5 years,	
	R11: a primary school teacher/cultural assistant; experience: 7 years	
	R12: a primary school teacher/cultural assistant; experience: 4 years	
	R13: a primary school teacher; experience: 2 years	
	R14: a primary school teacher; experience: 6 years	
	R15: a secondary school teacher; experience: 7 years	

This research project took place between November 2022 and June 2023. The sessions were conducted in Polish to allow the free flow of the respondents' ideas. The data collected in this way were recorded, transcribed and translated into English, after which followed their analysis. The analytical framework implemented in our data analysis rests on selected assumptions and tools originating from SA.

### 3.2 Data analysis framework: situational analysis

Situational analysis centers on examining the specific situation under scrutiny, encompassing all the components therein, including both human and non-human/technological/infrastructural elements, as well as the complex interactions among these components (Clarke et al., 2022, p. 5). This method seeks to offer a more profound insight into the situation, which can be highly beneficial for practical social applications. Research conducted through this approach takes into consideration the fluidity of connections among diverse entities and the uncertainties surrounding these connections. It recognizes that circumstances are perpetually changing, and analyses are inherently limited in scope, and bound by time (Clarke et al., 2022, p. 7). A pivotal concept in this methodology is the “situation,” distinct from the concept of “context,” which encompasses the interrelationships among the various elements within a specific temporal and spatial setting (Clarke et al., 2022, p. 18).

We have embraced this approach because SA highlights the “agency of the situation itself.” In our particular case, this unprecedented situation involves a multitude of factors, including the school as an institution, the targeted groups (comprising teachers and migrant pupils who are not proficient in the language of schooling), and the emergence of circumstances that necessitated the actions of teachers, such as the sudden increase in the number of newly arrived Ukrainian pupils due to the military conflict. SA is especially advantageous for our exploratory analysis due to its foundation in the principle of critical interactionism, which acknowledges variations in perspectives, commitments, loyalties, which influence social life at both individual and collective levels. This analytical approach also underscores the significance of “epistemic diversity and inclusivity in research” (Clarke et al., 2022, p. 9) and actively listening to marginalized or less-heard voices to advance social justice (Clarke et al., 2022, p. 20). Immersing deeply in the available data allows researchers to move beyond privileged interpretations and incorporate the voices of disadvantaged or overlooked groups. Within this framework, reflexivity becomes an essential trait for researchers to comprehend the intricacies, including various positionalities and differences.

We have generated all the maps recommended by SA, including situational, relational, social worlds/arenas, and positional maps. However, due to space constraints, we have chosen to present the ordered situational map (Clarke, 2005), as it proved to be the most effective in illustrating the interconnected organizational and institutional elements within the specific situation at hand.

## 4 Findings

In this section, we present findings of our research following the Ecological Systems of Human Development as conceptualized by Bronfenbrenner (1979, 1992, 2005) and discussed in Section 2.2.

### 4.1 Microsystem - individual and personal factors

#### 4.1.1 Previous exposition to linguistic diversity: experiences and beliefs

As already stated, before February 2002 linguistic diversity in Polish schools was almost non-existent. Except for schools offering preparatory classes, the majority were predominantly monolingual. As a result, few teachers had prior experience working with pupils with a migration background (PMB). In our study, some teachers reported their experiences related to teaching children of Polish origin who had returned to Poland after residing in the UK. These children, who were either born or had spent most of their lives in the UK (IR4), caused unique challenges. In 2022, most of the newly arrived PMBs were, however, of Ukrainian descent, with some exceptions, such as those from Belarus, Kazakhstan, Georgia and Vietnam.

What set apart the pre-war migrants was their deliberate choice to relocate to Poland, usually for economic reasons. Such decisions typically involved certain preparations for the stay in Poland, along with acquiring some proficiency in the Polish language prior to the relocation. Furthermore, these migrants exhibited high levels of motivation to learn Polish and integrate into society. Comparing the pre- and post-war contexts, our respondents drew the following observations:

*Overall, it was completely different. There weren't as many people - there were individuals, but it wasn't as visible as it is now. So that has changed (IR 3)*.

*About 20 per cent were already there before; usually, these are children from older classes ... and 80 per cent only arrived after the war broke out (IR5)*

When it comes to personal capacities (skills and knowledge, educational background), most of the interviewees declared themselves not to be prepared to teach in multilingual classes. This sense of the overwhelming unpreparedness of the whole teacher population for the contingent situation, exemplified by the excerpts below, was not correlated with the participants' career length:

*No one's ready, and we're just improvising it as we go (IR 3)*.

*I think that as teachers – and I'm not just talking about my school, but as a professional community – we are completely unprepared (FG1, R2)*

*As for whether the school is prepared, [3.0], I'll answer briefly: it's not (IR4)*

Teachers declared that this was mostly due to insufficient education received during their teacher training. This is supported by Szczepaniak-Kozak et al. (2023, p. 44–51), who claim that university curricula for teacher education in Poland do not offer language-sensitive modules and, with few exceptions, completely ignore this aspect of the teaching profession.

*Back in my time, this wasn't available, and I also received a clear answer, i.e., that universities currently don't acknowledge this need. It's not included in their programs (...) the system doesn't seem to notice it, and these young teachers will come in unprepared, banging their heads against the wall again, perpetuating the cycle (FG2, R4)*.

*No one studying biology, physics, geography, or any other major is taught how to simplify content. I believe we should deplore this tremendously (FG3, R4)*.

This lack of preparedness might have evoked teachers' initial concerns about the pupils' proficiency in Polish (the language of schooling) and about assessment tools and criteria.

*Having admitted non-Polish speaking students, there is a lack of assessment guidelines, a lack of guidelines (FG1, R2)*.

*Well, I am also thinking about the primary school-leaving exam (FG1, R2)*.

The respondents unanimously voiced a need for professional development to enable them to give a better response to similar emergencies, peppered with the remark that they struggle with a huge workload, which causes them to lack time for such activities.

*Consequently, the truth is that every teacher should undergo postgraduate studies or at least a short course in teaching the Polish language, to get a feel for the fact that these specialized language terms aren't mere fiction; they're a living language that can be practically applied (FG2, R4)*.

With regard to teachers' beliefs, we could sense that their general and declarative attitude to the increasing diversity in Polish schools is positive. An example of a typical response is presented below.

*Cultural and linguistic diversity is always a positive phenomenon, no matter where it occurs, and at school even more so, because it always influences and arouses tolerance, and curiosity about other cultures, and other nationalities (IR4)*.

#### 4.1.2 Teachers' emotions in the crisis situation: first reactions and emergency steps taken

Given that the majority of teachers were not prepared to offer instruction in multilingual classes, the emergent shift to a multilingual school environment triggered intense emotions, usually a blend of shock and fear, as illustrated in the excerpts below.

*And I remember my* ***shock*** *when I went to my first lesson as a substitute teacher, where some people didn't understand anything at all and I didn't know how to conduct the class (FG1, R2)*.

*So, we found out during one of those staff meetings that our school was going to be the only one in the district with preparatory classes*. ***Mixed feelings all around****, you know. On the one hand, there was this sense of mission, especially from the principal. On the other hand, there were* ***worries*** *about whether we could handle it, especially with space limitations, since we were already running double shifts. So, all sorts of thoughts were buzzing around. It was really tough (FG1, R3)*.

*Even though, as I mentioned earlier, the school had some experience working with foreign students*, ***it was still a shock for everyone****. The biggest challenges weren't related to education at all because these children often arrived with nothing. Moreover, they carried a* ***war trauma*** *(FG1, R1)*.

*We were all* ***shocked****, and I didn't expect it to drag on for so long (IR3)*

The feeling of losing their grip on the situation was intensified due to the language barrier.

*They were warmly welcomed with great understanding. However, everyday life became somewhat of a challenge due* ***to the language barrier*** *(IR4)*.

In response to those unprecedented circumstances, some teachers initially decided to continue the normal working mode.

*The kids are just assigned to a class, and even the class teacher in the meeting said that the classes would be taught normally, and the kids just have to learn, that was the comment*.

Some others applied contingency solutions to reach out to the newly arrived refugee pupils, despite PMBs' lack of communication skills in Polish. Firstly, in some schools, there were a few teachers who attended school in the 1980s and were therefore able to communicate in Russian. They were asked to play the role of language brokers for others. However, the vast majority of the teacher population have never learnt Russian, let alone Ukrainian.

*Not being able to communicate with them, the situation was salvaged by older teachers who had some knowledge of the Russian language. Even before the special classes were established, before the additional teachers and translators arrived, these teachers proficient in Russian supposedly attended classes acting as intermediaries. However*, ***the tragedy*** *was most apparent (FG1, R1)*.

Only one of the teachers (IR7) surveyed by us used materials offered by Ukrainian educational authorities to help the situation.

*The Ukrainian Ministry of Education has posted online textbooks in Ukrainian on its website for children who* ^***^ *would like to undertake this form of teaching. So in my class, it was possible to* ***use*** ***some of the illustrations or exercises posted there****, which the students then did (IR7)*.

Apart from an intensive Internet search for adequate materials, some teachers decided to learn the basics of the Ukrainian language to communicate with newly-arrived pupils. As one kindergarten teacher reports, she learned some basic expressions in Ukrainian by herself and taught them to the preschoolers in her group so that they could welcome their new groupmates.

*I learnt basic vocabulary in Ukrainian [...]. I also taught it to our kids, because they were very eager to learn and we always greeted... we greeted our new groupmates just in Ukrainian (IR6)*.

Her engagement activated the Polish children's agency; they were eager to learn new vocabulary in Ukrainian and asked what else they could do to help (IR6). The kindergarten children even took the initiative to teach their new colleagues some Polish and were highly motivated to communicate with them. Both parties seemed open to their multilingual experiences and their different first languages did not seem to erect a barrier:

*When we went out for a walk, they would also tell their new friends what they found in the area, and they would mention various anecdotes to them. Well, sometimes I wondered to what extent these Ukrainian children understood what they were saying to them, but neither party seemed to bother (IR 6)*.

To facilitate more successful learning, most teachers translated classroom materials (worksheets) into Ukrainian or Russian, usually by online translators (e.g., IR3). They also attempted to adapt their regular materials, following their intuition. Although this is a very good example of teacher agency, or rather their contingency competence, the materials presented to us during the interview sessions indicated a general lack of knowledge about teaching in a language-sensitive manner. Some good practices which could be observed in the material presented during our interview sessions were: asking comprehension questions, simplifying the language used in the original materials in Polish, frequent repetition and drawing on non-linguistic resources, translanguaging and allowing Ukrainian pupils to take oral instead of written tests (e.g., IR7). Teachers in kindergartens, where pupils do not read and write, prepared posters with pictures representing basic needs which they might have, e.g., going to the toilet or feeling thirsty. Thanks to this, children could communicate their needs by pointing at an adequate picture (e.g., IR6, IR7). Creating a stress-free atmosphere and providing language props were also present in primary schools.

Finally, most teachers declared that in response to this emergency, they participated in training sessions offered by various entities such as NGOs, teacher training centers, universities, or even individuals. Because the military conflict overlapped with the social contact restrictions caused by the pandemic, these took the form of webinars organized on the spur of the moment as grassroots innitatives. The agendas of these events covered diverse topics, including the basics of the Ukrainian language, teaching-material development, and psychological counseling, especially in response to the Ukrainian pupils' traumatic experiences of the war.

*And just the fact that teachers have started to receive training in this matter. That's something very important (FG2, R3)*.

In sum, at micro level, teacher agency triggers encompassed individual initiatives and uninformed approaches prompted not by professional development or ambition, but rather by the urgency to address an emergency or to manage a crisis. These responses often stemmed from emotions such as fear, and compassion toward Ukrainian children and their families. In the first weeks following the invasion and the influx of Ukrainian pupils to Polish schools, the main concerns included overcoming the communication barriers and settling the children into the new educational system. Most of the school staff's efforts were thus focused on the pupils' integration and learning of the Polish language, rather than maintaining pupils' linguistic capital.

### 4.2 Mesosystem - school environment and a network of relationships

At this level, our analysis concentrates on two main aspects of teacher agency: Material factors (resources, physical environment) and Structural factors (intra-school cooperation groups especially for materials exchange and translation help, roles, power and trust).

#### 4.2.1 Material factors

Because Polish teachers had at their disposal limited didactic resources, they almost instantly took grassroots initiatives to prepare materials adjusted to the pupils' special linguistic needs. In the face of the fact that they were offered practically no training support in this regard, the materials were created individually or in cooperation with other teachers, following their professional intuition rooted in experience. All teachers complained that it took a considerable amount of time to prepare the materials.

*I* ***tried to translate initially from Ukrainian into Polish****, or vice versa, the instructions, or short pieces of information about what the lesson would be about. But this was simply a* ***big effort*** ***for me and took up a lot of time****, and* ***I was not always able to prepare such materials*** *for these students* ^***^ *(IR7)*

*My colleagues and I also discussed what we could do. ... Well, once we prepared the first materials, it went downhill (IR 6)*.

Drawings and illustrations were used frequently. Some teachers also noticed that it was easier for Ukrainian learners to make graphic notes, especially when they were Russian-speaking and thus using the Russian Cyrillic alphabet. Moreover, instead of teachers translating texts for pupils, it was more effective to allow pupils to use online translators themselves – a better quality of translation was achieved this way. This was particularly important during tests, when a proper understanding of the rubrics was necessary (e.g., IR7; FG6, R2). As some teachers reflected, these aids turned out to be equally helpful for Polish learners in their classes (sic!). This is illustrated by the account of a biology teacher who talks about her experience concerning material development.

*So we had to start learning slowly, we just had to create materials that were accessible to these children as well. Suddenly*, ***it turned out that you can teach biology very well with*** ***pictures*. ***You don't need to have a textbook and two A4 pages of texts, which are scary also for Polish children, because there are a lot of difficult words, but* ***suddenly it*** ***turned out that it can be done in a much easier way, more accessible*** *(FG2, R4)*.

Other language-sensitive practices reported by teachers were: bilingual signs on school premises, Ukrainian textbooks and other books in school libraries, cards to communicate with teachers with most common expressions in Polish and pupils' first language(s).

#### 4.2.2 Structural factors: intra-school cooperation groups

Teacher agency goes beyond the individual level when teachers take collective responsibility and begin to build their professional capital together. This type of agency is also visible in our data. Quite a few of our respondents mentioned that probably the most appreciated aspect of this crisis situation was the emergent network of relations and a greater sense of community, which consequently triggered individual agency. Earlier, teachers did not share materials or hold extended discussions about their teaching matters, usually due to lack of time. In these circumstances, they felt they could rely on one another and this motivated them to work even harder, e.g., offering translation help.

*We talked to each other about it in the staff room*, ***supporting each other****, trying to understand the situation somehow, and we did the same thing on the first few days in lessons (IR4)*.

*As I know Russian, somewhere in there I was also* ***trying to facilitate the work of other*** ***teachers****, those who did not know the language. When they wanted to talk to those students there, well, I was also present as an interpreter (IR3)*.

In a few cases, teachers or school principals liaised with other schools in the neighborhood (IR1), seeking opportunities to become more knowledgeable and prepared for the new situation. Sometimes Ukrainian pupils, especially those who arrived earlier, or community interpreters (usually parents) were asked to act as language brokers.

*There was also a Polish student sitting next to them, who provided support and explained during the lesson if someone did not understand something (IR8)*.

More significantly, teachers started to appreciate how important these professional relations and mutual trust among teachers are.

*Therefore, creating these materials in collaboration with other teachers, exchanging these experiences, is something really great. Finally, I noticed that I'm not alone as a subject teacher; there are other teachers with whom I can now exchange these experiences, so that's definitely a huge plus (FG2, R3)*.

Additionally, online groups and fora were spontaneously created where teachers supported one another and exchanged materials or useful information. Apart from the professional assistance, these online spaces were very much appreciated for their role in building a sense of professional togetherness and chances to communicate with other teachers in the extended community. It seems that there is a greater need for such occasions and spaces, which is something school management could take into account.

*But I think that the* ***strongest source of information exchange among teachers remains*** ***that unfortunate Facebook*** *and those online teacher groups of biologists, non-biologists and so on. [...] after the outbreak of the war, and the teachers started to share materials, discuss things*, ***it was so cool that we teachers wanted to do it*. ***[...]* ***Teachers started talking to each*** ***other*** *(FG2, R3)*.

With regard to the school management support and its impact on the teachers' agency, it needs to be said that most of our respondents were rather dissatisfied with the administrative assistance they received (e.g., IR5). In their opinons, they were left to themselves, did not feel supported by the education governing bodies, and did not receive any teaching materials. Some incidental support, not coordinated by educational authorities, was offered by a few teacher training centers. Additionally, commercial publishing houses sent teachers some teaching materials or organized training webinars.

*I think that systemically, there was* ***absolutely no preparation or support*. ***[...] So unfortunately this lack of preparation is coming from the top and actually a lack of support (FG1, R3)*.

*Generally, this all hinges on us*. ***I didn't receive any such help from the school*.** *However, the publishing house is there to help. You can sign up for free workshops, and some online meetings (IR3)*.

***We could only support each other*** *and [3] in whatever depths of the Internet trying to find anything to be able to somehow work with these students (IR4)*.

Despite the hardships, teachers appreciated these experiences and felt empowered, as, generally, they managed to deliver successful instruction. There were numerous occasions for them to reflect on their teaching practices, especially those in multilingual classes. They gained very precious expertise in pupils' integration into the school environment, for example, allowing a silent period for them, the importance of a gentle and patient approach to children traumatized by the war, and the benefits of learning in multilingual classes. One of our respondents mentioned that kindergarten children adapt more easily and that the arrival of non-Polish pupils was beneficial, because the whole group became more open to other cultures and languages. The pre-schoolers also acquired some vocabulary in other languages, including (unfortunately) swearwords (e.g., IR6). The same respondent (IR6) further noticed that communication is possible even if children speak different languages. These experiences enabled teachers to develop a calmer, almost fear-free, attitude to their daily practices, empowering them as professionals.

With regard to the downsides of the new circumstance, the transitory character of migration and relocations appeared as a recurring demotivating theme in the data. Numerous respondents emphasized the negative impact the unstable life situation and relocations have on children, especially teenagers who are tired of, for example, being suspended between the two educational systems (Polish and Ukrainian) especially in the final classes of primary school (FG2, R3) when school-leaving exams take place. The excerpt below exemplifies what was repeated in the teachers' responses.

*It looks as if* ***they study with us and come home and still have a second school there*.** *This also affects the quality of how they work with us. These children are overtired, demotivated and it's also hard then to arouse this mechanism as if learning the language, this motivation (FG2, R3)*.

There were also some voices calling for the revision of school curricula that impose learning two foreign languages (e.g., English and German/Spanish). Taking into account that Ukrainian pupils also need to master Polish, which is a considerable burden, our respondents suggested that their first languages should be recognized and qualified as one of the obligatory foreign languages in the curriculum (FG2, R1). This would allow the pupils more time to learn Polish. The value of preparatory classes or additional classes in Polish as a second language has also been considered by our respondents as an important element of successful whole-school integration (e.g., FG2, R3; IR5).

Some respondents also pointed to a significant disparity in attention and support allocated to Ukrainian war refugees and other children of East-European descent in comparison to children from other countries or backgrounds. Notably, the latter group might face more challenges related to a heightened language barrier, rendering communication and their whole school experience more arduous and leading to their being almost neglected in receiving adequate focus and assistance.

*For this reason, when thinking about these textbooks, it would be worthwhile to consider making them truly universal, so that they can be used with a child from any part of the world*, ***so that it is not just a textbook dedicated to Slavs*. ***They have it much easier anyway as if they had already come here with something, with some of their linguistic capital, which is easy for them to convert into our realities. On the other hand, Georgians, children from Bangladesh, and children from Africa face incomparable challenges. In fact*, ***after a few*** ***weeks, some teachers pretend not to see such children*. ***However, they are here, they want to be here and we have to make it possible for them in some way too (FG2, R3)*.

One teacher also mentioned that Polish pupils cannot be neglected in the entire integration process either.

*Schools send their children to us, we don't complain. But on the other hand*, ***it's also a*** ***school for Polish children*, ***and we can see that it doesn't quite fit together (FG2, R3)*.

Apart from linguistic barriers, our respondents also call for diagnosing children's special educational needs. Because migrant pupils come from different educational systems or relocate several times, their learning or physical deficits may go unnoticed.

*They also often* ***need to be diagnosed*** *because, after all, these are also children with different deficits. We kind of see it, and there are no tools to help us in any way (FG2, R3)*.

The last element of the intra-school level we want to discuss is the way the teachers talked about their lack of agency when it comes to the psychological support they can offer to the newly arrived pupils and how they coped with the distress caused by the new circumstances. Acting upon intuition and humanity, they usually remained in waiting for the children to feel better and resorted to patience and gentleness.

*These children have gone through trauma, yes. [...]* ***We*** need^***^to wait *for them to open up*,^***^ *to trust someone again (IR3)*.

*We were all very open, we had a lot of* ^***^ *patience* ^***^ *to reach an understanding with these individuals because there were often moments when we would say something to them, and they would look at us without a response. There was a lack of reciprocal communication, so* ***we were very patient*** *(IR3)*.

Their agency revealed in their attempts to learn some polite phrases in Ukrainian for children to feel welcome in the new school groups.

*We also tried to make sure that the children learned a few phrases of politeness in Ukrainian, so that the reception of children traumatized by war situations would be as warm as possible (IR7)*.

Only rarely did the teachers ask for consultations with psychologists employed at schools. One novice teacher reported that she sought advice from a psychological counselor, which enabled her to set “goals and how to approach them” (IR3). Finally, one teacher reported that in her school, a specialist in pedagogy and psychology organized joint sessions with teachers and migrant children, to which parents were invited as well. They enabled teachers insights into the refugees' plight and everyday situation, but in our study this was an isolated case.

*[a] pedagogue-psychologist who spoke Russian ... and he at the beginning, it was immediately April-May somehow and he had cyclical meetings with the families of the children .... that is, with the parents, as well as with the pupils themselves, and I know that this took place ... periodically, every week (IR5)*.

What this section reveals is that the crisis situation enacted the structural aspect of teacher agency and pointed to the value of building professional relationships and cooperation. Despite hardships and lack of governmental support, our respondents highly appreciated collaborating with their colleagues and sharing their experiences, practices and materials. This newly-emerging sense of collegiality empowered teachers and motivated them to work.

### 4.3 Exosystem: teacher-parent cooperation

Now our attention turns to those aspects of teachers' structural agency which stem from their interactions with parents, especially those initiatives which school personnel, teachers included, undertook to integrate parents into the school ecosystem.

All schools held integration events to which Ukrainian pupils were invited with their parents (IR5). Parents in general were encouraged to contribute to schools' functioning by, for example, helping with events such as seasonal decorations, food for school festivities or, as mentioned earlier, working as volunteer community interpreters for other parents (IR1–10).

*Parents of Ukrainian children also prepared additional decorations, yes, baked foods for the Christmas fair (IR3)*.

Some schools showed a more inclusive approach by consulting Ukrainian parents about their own and their children's needs.

*At our kindergarten, there was a brainstorming session [with the parents] on how to organize it all in the best way, ... how to introduce the children to the group. YY. The parents offered tips on what the children don't like, what they like, what to do (IR6)*.

In the process, it frequently appeared that there are also language barriers in communication with parents.

*[t]he language barrier is a problem for how to activate parents on school premises in any way (IR4)*.

Where (community) interpreters were not available, teachers reported that email communication is preferred because parents have more time then to read the message, and for example, use an online translator.

*[m]any of them prefer e-mail contact, where they can use an interpreter in the calm of their home and express what they want to communicate (IR3)*.

More importantly, some of our respondents noticed that not only children but also parents need support, including psychological help, and that cultural assistants can play a significant role in this area (FG2, R2). Some teachers see school-home cooperation as a means of preventing future problems, especially due to the legal and cultural differences in parental responsibilities.

*Hopefully, there are no serious problems among the children, thanks to us, our vigilance, the fact that every action that shouldn't be taken is immediately explained to the parents, and the fact that we've also had a lot of preventive meetings this year*, ***explaining the mysteries of*** ***Polish law to the parents*** *who are often completely unfamiliar with what a civil servant means in our country and what a minor is responsible for, and* ***what a parent is responsible*** ***for*** *(FG2, R3)*.

When it comes to problematic aspects of this type of cooperation and ways of coping with them, despite access to the internet and opportunities for relevant training, Ukrainian parents fail to use online communication tools with school and with other parents in the class, which is the standard means of communication in Polish education. One teacher voiced her exasperation with this situation, accompanied by quite a few reflections about its causes, that is, whether it is caused by inhibition to write in Polish or lack of interest on the parents' side (e.g., FG1, R2). Our respondents also mentioned that some stay-at-home moms tend to be very involved in their pupils' school life. While their nurturing approach brings certain benefits, there were cases where their expectations exceeded what the school could provide. Notably, a critical concern emerged when some of these moms intervened in children's interpersonal conflicts. In certain cases, their involvement escalated to verbal or, at times, physical abuse toward other children. Such behavior is deemed unacceptable in Poland, particularly on the school premises, where the school personnel hold legal responsibility for the safety of all pupils.

Finally, it is worth taking into account cooperation with Polish parents. As one of our respondents says, some parents reacted with distrust to the new composition of classes which their children attended. They considered the classes too big, almost “occupied.” With time, the situation became more tolerable for them, but fewer parents remain eager to help Ukrainians.

*There were already such voices in the spring when we had these preparatory classes. Polish parents reacted very negatively:* **“*Another collection? The school is overloaded, the*** ***teachers are tired, it's another shift, the classrooms are occupied”*. ***Now I no longer hear such voices. There are fewer Ukrainian children because those from outside the region have gone to their own schools. The Ukrainian children have integrated a little bit with the rest and it seems to me that this effect is no longer there (FG1, R3)*.

In summary, our respondents consider pupils' parents to be essential elements of the school ecosystem. Teachers have made efforts to integrate and engage parents in their children's school life. This task, however, was not always easy due to certain cultural differences such as, for example, different models of parents' school engagement, child-raising patterns, along with language barriers or limited availability of parents, often overwhelmed by the hardships of settling in a new country. Despite these difficulties, the teachers were proactive in integrating parents of newly-arrived pupils and responsive to concerns raised by some Polish parents, sometimes addressing conflicting needs.

### 4.4 Macrosystem: national level

At this level, we delve into teacher agency as dependent on extended context factors, here support, or lack thereof, from regional and national authorities responsible for education in Poland. For lack of space, we do not analyse societal factors which bear influence on teacher agency.

In general, our respondents expressed an immensely negative evaluation of the regional and national level support offered in this contingency. They felt that there was no interest on the part of the national governing bodies in the situation and development of systemic solutions. If some efforts were visible, they were limited to allowing more preparatory classes with no interest in providing means for their adequate functioning (FG2, R1). These sentiments are illustrated by the excerpts below.

***We have not received any support*** *from either local authorities, national authorities or any non-governmental institutions (IR4)*.

*Additional measures should be taken, but unfortunately, there are no such measures (IR 3)*.

One of the respondents noticed a positive change, namely an increased number of Polish classes:

*Well, perhaps the one positive thing that has happened, from my perspective, is that the* ***number of maximum hours in these classes has been increased from 5 to 6*, ***which from my perspective as a Polish language teacher […] is beneficial. The children are overloaded, and this 1 hour can always be devoted to something else, to some extra play or other things (FG3, R3)*.

There were also voices mentioning that the situation was worsened by trivialized and neglected financial issues, for example, lack of gratification for teachers working in difficult classroom conditions, where children with special education needs were pooled with children not speaking Polish. Furthermore, financial rules differed across the country (FG2, R1), which teachers considered as demotivating. Instead of concentrating on their daily work, they wasted time and energy on seeking legal loopholes. In such circumstances, the organizational and financial support offered by UNICEF was evaluated as very helpful (FG2, R3).

The teachers also offered reflections on what could help them to perform better. Firstly, they saw a great demand for systemic psychological support for pupils and parents in Ukrainian and their other first languages. They rightly claimed that it is almost impossible to help a war-traumatized child in a session led in Polish via an interpreter, and such sessions are less successful in the presence of a third party. Enabling Ukrainian-speaking psychologists to be registered professionals in Poland would help this situation immensely, especially in the face of serious psychological problems reported by the teachers, including suicide attempts (e.g., IR9). In teachers' opinions, Ukrainian cultural assistants are not qualified to offer this type of counseling (e.g., FG3, R1).

The schools, in general, call for more cultural assistants, because their number is still insufficient (FG2, R2). They can play not only the role of community mediators but also can enable pupils to be able to report their needs and difficulties in their first language(s). This can have a calming effect on the children.

*A child in the class said “Oh good, here at least one person speaks Ukrainian” (FG3, R1)*.

*[c]hildren are tense and for them*, ***it's important to talk in their own language. [*…*] the*** ***brain relaxes and the learning goes better*** *(FG3, R1)*.

Secondly, our respondents called for more preparatory classes. The ones already functioning tend to be overcrowded, which makes promoting a quick transition to regular classes a necessity. In a similar vein, there is a huge demand for textbooks designed with a focus on the specific needs of pupils in preparatory classes, which could greatly facilitate the process of teaching and learning Polish. The existing textbooks for Polish as a foreign or heritage language are not written to enable a more successful acquisition of Polish as a language of school instruction.

To sum up, the respondents were deeply disappointed with the lack of institutional support. This disillusionment, also drawing on their previous experiences, did not, however, evoke a sense of helplessness. Instead, it enacted their agency and motivated them to face the challenges by themselves.

### 4.5 Chronosystem: the current situation

In this section, we discuss these aspects of teacher agency which are projective in character, both in the short/current and long-term perspective. It focuses in particular on the respondents' evaluations and emotions to the new situation in schools, and whether their emotions and attitudes changed over time.

One of the positive durable changes our respondents mentioned was that they feel more empowered as professionals. The fact that they withstood the emergency circumstances and continued effective teaching enabled them to be a bit more optimistic for the future, which is partly thanks to the positive attitudes which their pupils showed in contact with those newly arrived. Furthermore, the stress induced by the unprecedented circumstances has lessened and teachers feel more confident and stable (IR5). This is accompanied by a generally smooth integration of PMB in Polish schools. School populations have got used to more Ukrainians and their first languages in school corridors (FG1; R1).

*If I had a child speaking another language now, I would already know how to proceed*, ***I*** ***feel much more confident about it*. ***I've also seen that* ***the children are very cooperative*** *and also try to make sure that their new colleagues don't feel uncomfortable (IR6)*.

On average, the pupils' adaptation is declared to be going well. Initially, most of the children's first reactions were shock, astonishment and fear (IR4). It was also difficult to reach out to them because they were inhibited, also due to the language barrier (e.g., IR3, IR5).

*He [a pupil] came to us in ... in the middle of the school year*, ***he didn't actually*** ***make a single sound with his voice*, ***say anything until the end of the year (IR5)*.

A year later, teachers noticed great progress in the school atmosphere and the pupils' wellbeing. They see more trust and signs of progressive integration of the school communities.

*One pupil was very* ***frightened at first*. ***He, as we were addressing him*, ***would turn*** ***away or cover up, cover his face*, ***yes. He would wear a hood because he was just* ^***^ ***scared of this new environment*. ***When they went on a trip, this pupil didn't* ^***^ *know how to find himself, yes. He didn't even want to eat the lunch he got because he was so scared of the situation*. ***Today, he is a completely different person*** *[...]*. ***They are now getting on*** ***well*. ***They take an active part in the classes, they are already speaking more and more Polish* ^***^ *in a communicative way, also it is definitely better now, also* ***this change is really visible*** *(IR3)*.

Some of the Ukrainian children's proficiency in Polish gained in a relatively short period of time seems really impressive. As one of our respondents (IR9) – a teacher of Polish at a secondary school – reports, one of her Ukrainian students wins province competitions in reciting Polish poetry and the other excels in writing essays in Polish. This adds to both teachers' and pupils' willingness to work harder to improve the situation even more (IR3, IR9, IR10).

Nevertheless, more than 1 year after the contingent admission of Ukrainian pupils to Polish schools, on average, negative evaluations outweighed positive ones. This is so due to numerous reasons. Firstly, comprehensive materials for teaching Polish adapted to the school curriculum are still unavailable. Teachers continue using their own resources and express the need for visually attractive textbooks, since pupils do not find photocopied materials interesting. Importantly, the preparation of didactic materials remains uninformed and uncoordinated, which raises certain doubts related to their usefulness. Despite a certain availability of webinars, teachers still lack clear instructions on how to adapt their teaching materials for them to be universally used in class and comprehended by all pupils, not only those with Slavic backgrounds. Their agency is hindered by the feeling that they are not professional and, as one teacher says, the pupils are like guinea pigs in the entire process (IR9). They continue to design materials based on their intuition.

*From my point of view, what else would be useful? I think that in relation to this very*, ***a*** ***very large number of teachers*** *who are rushing to create these materials*, ***are somewhere*** ***very much in the dark*. ***I think that, to a large extent, it would be good to create some kind of a nice resource of guidelines for teachers*, ***to teach teachers how to create*** ***simplified materials*. ***We play with it, we try it out ourselves. We are just testing it on our students*. ***Do these materials make sense, are they understandable?*** *(FG2, R3)*.

Additionally, a year later, few or no changes are noticeable with regard to teaching Polish as a language of instruction or procedures applicable to placing pupils in preparatory classes. Voices are calling for more concentrated efforts and activities on the part of the bodies governing education, based on a thorough reflection of the now-functioning solutions. One of our respondents also raises the issue that some procedures are detrimental to pupils' integration: when they are considered as able to attend mainstream classes, they need to change school because only those pupils who reside in a particular district can attend this school. They need to change their peer group and adapt to a new environment (e.g., FG2, R1).

As far as teachers' wellbeing is concerned, our respondents often declare they feel tired, overwhelmed or even disillusioned.

*[i]t's like the principal teacher proposes something there and says “you can, you don't have to.” [...] and I think “Gee, now I have to waste another three afternoons because I have to learn Ukrainian”. [...]* ***it was more of a demand again on the teacher*,** *that”'well, do something with yourself' to make it look like something (FG1, R3)*.

*If I were a junior teacher in terms of seniority, maybe I had some hope that maybe the next education minister, that maybe the next government ... that maybe something will change (IR4)*.

Together with these emotions, there is fatigue caused by the prolonged crisis, with some teachers feeling burnt-out. This is accompanied by more teachers being concerned about Polish pupils in their classes.

*[w]e don't have time for the Poles anymore (IR1)*.

Our respondents reported that Polish pupils and their parents are also becoming more frustrated with what they consider unjust or unequal treatment of their children, in comparison to Ukrainian ones.

*There is definitely* ***more impatience among parents and some children*. ***I don't always experience it, but I also hear my colleagues talking about it (FG1, R2)*.

*Yes, it's the same with us (FG1, R3)*.

*They don't want the children to lose out on it. Sometimes the* ***children are jealous:*** “*Why can they do something and we can't?”. You have to choose your words very carefully, because* ***concern for foreigners may not always be well received*. ***It can be perceived as facilitation and injustice (FG1, R2)*.

In general, teachers swiftly responded to a crisis which enacted their agency in the short term – they were mobilized to seek information, learn, collaborate, and adapt their teaching practices to address newly-emerged challenges, primarily the language barrier. However, in the long run, there appears to be a sense of demotivation among them. They continue to draw on *ad-hoc*, makeshift solutions rather than develop and refine their newly gained skills and knowledge. Instead of aspiring to become well-versed in handling multilingual classes, they often perceive their current competences as adequate, given their survival through unprecedented contingent circumstances, while anticipating a return to a “normal” situation.

## 5 Concluding remarks

The findings confirm a dramatic transformation of the predominantly monolingual school environment in Poland and demonstrated to what extent the home languages of newly arrived pupils are manifested and fostered in the school environment. Our study provides evidence that Polish schools are at the early stage of multilingual education. Teachers' own perception of the newly-emerging linguistic and cultural heterogeneity determined the ways in which it is manifested in the school and home environments, and the extent to which possibilities for synergies exist between the two. The study also revealed how these novel circumstances are perceived by teachers.

In order to articulate more vividly “the elements in the situation” and analyse “relations among them” (Clarke, 2005, p. 86), and to answer RQ1 (and partly RQ2 and RQ3), below we present an Ordered Situational Map (Table 2). This map structures our findings and provides insight into the nuances of the crisis situation under study, which enacted teacher agency.

**Table 2:** Ordered situational analysis of a crisis situation enacting teachers' agency.

*Individual human elements/Actors: e.g., key individuals and significant (unorganized) people in the situation, including the researcher*	*Non-human elements actors/actants: e.g., technologies; material infrastructures; specialized information and/or knowledges; material “things”*
Teachers and cultural assistants	Educational system and legislature
School administration staff, including school principals	Teacher training system
School psychologists and pedagogues	School organization, e.g., preparatory classes (or lack thereof)
Pupils	Existing infrastructure, e.g., division into district schools
Parents	Teaching materials (and lack thereof)
Experts and teacher trainers	Information technology, e.g., social media, Internet fora
	Financial resources (or lack thereof)
*Collective Human Elements / Actors, e.g., particular groups; specific organizations*	*Implicated / Silent Actors /Actants, ss found in the situation*
Local community	Pupils' family members
Neighboring schools	Politicians / policy-makers
Formal and informal teachers' networks (e.g., group works, social media groups)	
Teacher training centers	
NGOs	
Government institutions at all levels (local and central): boards of education, local authorities, regional authorities, central government, ministry of education, etc.)	
Academia	
Other organizations, e.g., UNICEF	
*Discursive constructions of individual and/or collective human actors*	*Discursive constructions of nonhuman actants*
Teachers: resourceful, creative, hard-working and eager to develop themselves professionally despite the worrying/contingency situation	Incompatible school curricula (Polish and Ukrainian)
*we [teachers] are completely unprepared*	Lack of teaching materials
Lack of organized, institutional support, a sense of being unsupported, left alone, tired	Lack of clear instructions and regulations
*It is all on us [teachers]... as always*	*we don't know what to do*;
*the government doesn't see us [teachers]*	*we are in the dark*
*we're on our own*	
*teachers are tired*	
Unhelpful governing bodies, ignoring financial needs of schools	
*We have not received any support from either local authorities, national authorities or any non-governmental institution*	
Refugee pupils, still in transition, not sure of their situation, traumatized by war experiences, but eager to adapt	
*Frightened*; *scared*; *adapt quickly*	
*Political/Economic Elements, e.g., the state; particular industry/ies; local/regional/global orders; political parties; NGOs; politicized issues*	*Sociocultural/Symbolic Elements, e.g., religion; race; sexuality; gender; ethnicity; nationality; logos; icons; other visual and/or aural symbols*
War context	Monolingual ideals, especially neglecting the worth of the entire linguistic capital of pupils and concentrating on teaching Polish as the language of instruction.
Increase in numbers of non-Polish pupils	Linguistic diversity seen as a challenge rather than an asset
Governmental response to the crisis situation	Mononational / monocultural mindsets
National bias/ animosities originating in historic conflicts	Xenophobic prejudice
Economic concerns (extra costs related to accepting refugees)	Yearning to “get back to normal”
	Different school cultures and legal regulations in Poland and Ukraine
	Different approaches to parenting
*Temporal Elements, e.g., historical, seasonal, crisis, and/or trajectory aspects*	*Spatial Elements, e.g., spaces in the situation; geographical aspects; local, regional, national, and global spatial issues*
Working with multilingual classes, preparing materials considered time-consuming and an extra burden	Distribution of pupils in classes, schools and in districts
Huge effort / extra workload	Integration issues (e.g., pupils' placement in preparatory classes vs. mainstream classes)
Overtime issues	Overcrowded schools and classrooms
Invisible aspects of teachers' work	Transition from a mono- to a multilingual school landscape
Changes in emotions and evaluation of the contingency situation over time	
*Major Issues / Debates (Usually Contested)*	*Related Discourses (Historical, Narrative, and/or Visual) e.g., normative expectations of actors, actants, and/or other specified elements; moral/ethical elements; mass media and other popular cultural discourses; situation-specific discourses*
Too much focus on Ukrainian refugee pupils - other pupils with migration backgrounds not visible, neglected	Unpreparedness (of the state, the school system, teachers)
School performance-related concerns (e.g., school-leaving exam results)	Lack of governmental support
Huge involvement at the individual level – grassroots initiatives	Historical sentiments (unresolved conflict areas in the Polish-Ukrainian past raised by some nationalist groups)
Crisis fatigue	Anti-migration attitudes in society at large visible, e.g., in hate speech propagated in social media
The helper's stress / burnout	A shared belief in resilience and resourcefulness characterizing Poles and their ability to function well in crisis
*Other kinds of elements*	
Emotions of pupils, teachers and parents (shock, fear, trauma)	
Fatigue experienced by teachers (work overload), pupils (learning new languages; attending Polish and online Ukrainian-online schools), parents (often working overtime; overwhelmed by life issues)	
Establishing makeshift solutions and their persistence due to the lack of other solutions	
*The emergent is the new normal*	

In response to RQ2, our findings highlight that despite teachers' inexperience and unpreparedness for the new educational context, they instantly responded to the challenges that emerged, showing crisis management skills (cf. Erol and Karsantik, 2018). In the data, we have evidence of the immediate spontaneous grassroots response of the whole school community to welcome the newcomers through the symbolic presence of the Ukrainian language in the school linguistic landscape, as well as actual attempts to communicate with them in their home languages (Ukrainian and/or Russian). There were also instant manifestations of these minority languages in the school environment (posters, events, welcome packets). However, these were rather uninformed responses, leading to a superficial integration based on grassroots initiative, peer support, and a general willingness to educate. The teachers reacted following their intuition and previous experience. Their agency revealed in taking the initiative and ‘out-of-the-box' thinking, but a true integration of language-sensitive teaching did not take place. We could only find isolated instances of teachers' attempts to integrate the minority languages in the form of multilingual glossaries, using online translators, allowing more graphic aids or simplifying their teaching materials in Polish.

Practically devoid of any institutional state support and previous relevant training opportunities, teachers spontaneously made efforts to adapt their didactic materials and overcome the language barrier. Other examples of teacher agency in this contingency are their collaboration in material design as well as the willingness to participate in courses sensitizing to migrant students' needs, such as linguistic, educational, or emotional ones. Almost 2 years after the war broke out, there are not many teachers who are prepared or qualified to teach in such classes, and there is a scarcity of instructional materials in languages other than Polish (cf. Papasoulioti et al., 2023). The same needs to be said about activities enabling the integration of home languages into the teaching/learning process: language-sensitive teaching is not applied, and some teachers rely on contrastive demonstration or Ukrainian pupils' gained proficiency in Polish.

With regard to current perception of the situation and in response to RQ3, the initial enthusiasm seems to have worn off and the makeshift solutions have been widely accepted. Thus, the main challenge that remains is motivating the whole school community toward further development of their competences and multilingual whole-school development and streamlining the existing solutions to be prepared for working with multilingual classes and migration. Most of our respondents pointed to the fact that they “survived” in a crisis situation and still rely on makeshift tools and solutions they developed in the first month after the increased Ukrainian pupils intake. Some of our respondents experienced crisis fatigue – a kind of tiredness or burnout due to the continuing contingency situation. Maintenance of pupils' home languages is not well catered for because on average Polish teachers lack awareness, preparation, and focus on language maintenance support and often provide parents with badly-informed advice. Finally, more and more voices are raised that too much focus is placed on Ukrainian refugees and children of East-European origin at the cost of others, especially non-Slavic pupils who might find it more challenging to master Polish and assimilate.

On the plus side, what emerges from the data is that teachers started to appreciate the fact that more languages are present in schools and no longer treat this diversity as something to be afraid of. In the data collected, we could see evidence of our respondents' positive evaluation and appreciation of their pupils' multilingual repertoires. They reported situations in which pupils capitalized on them, leading to their better school performance, for example, in kindergarten. In the long run, there are chances for a “multilingual turn” (Conteh and Meier, 2014; May, 2014) in Polish schools. For this to take place, however divisive or political it may seem, the Polish education system needs to undergo a systematic change in the body of teachers hired. The school staff needs to become more linguistically diversified in order to mirror the diversification existing in the pupil population.

While this study provides valuable insights into teachers' responses to crisis situations and their enactment of agency, it is important to recognize several limitations inherent in its qualitative format. Qualitative research, with its emphasis on depth over breadth, may limit the generalisability of our findings to broader populations or contexts. Additionally, the utilization of a situational analysis approach, while offering rich contextual understanding, may constrain the transferability of the findings beyond the specific settings examined. It is worth noting that this paper represents an initial exploratory investigation into an unprecedented situation. A more comprehensive and methodologically diverse follow-up study is warranted. Exploratory studies, typically qualitative case studies like ours, lay the groundwork for further research. Future research endeavors could incorporate mixed-methods approaches or larger sample sizes to enhance the generalizability of findings and deepen our understanding of teacher agency in crisis situations. By addressing these limitations, a more nuanced understanding of this complex phenomenon could be obtained.

## Data availability statement

The raw data supporting the conclusions of this article will be made available by the authors, without undue reservation.

## Ethics statement

Ethical approval was not required for the study involving humans in accordance with the local legislation and institutional requirements. The participants provided their written informed consent to participate in this study.

## Author contributions

AS-K: Writing – original draft, Writing – review & editing. EW-F: Writing – original draft, Writing – review & editing.
