# Supplementary material for: Teacher agency in the times of crisis: a situational analysis of school environment after the 2022 Russian invasion in Ukraine
Source: Front Psychol. 2024 May 23;15:1382403. doi: 10.3389/fpsyg.2024.1382403 (PMC11153657; doi:10.3389/fpsyg.2024.1382403)
Supplement: Supplementary file 1 [file Data_Sheet_1.pdf]

Teacher agency in the times of crisis: a situational analysis of school environment after the 2022 Russian invasion in Ukraine

Interview/focus group script

1. **Introduction**
   - Introduce yourself and thank the respondent for participating in the study
   - Brief description of the study, assurance of anonymity, consent to audio recording
2. **Bio data**
   - sex
   - function in the school (teacher, cultural assistant, methodical advisor, principal);
   - if applicable, subject taught;
   - professional experience (in years)
   - type of school (e.g. primary, secondary) and its location (e.g. town, village); do not ask for the specific school number and name!
3. **Main questions**

3a. Linguistic and cultural diversity

- Is it present?
- Was it present before February 2022?
- How does the respondent perceive it - positively / negatively
- Were schools/school staff prepared for it?
- Crisis situation (first reaction and actions taken)
- How did schools/school staff react? What actions did they take, e.g. language preparation classes, school events?
- How did school staff/the respondent feel (e.g. helplessness, fear, frustration ...)?

3b. Elaboration of teaching materials

- Did they prepare any in response to the crisis situation?
- Did they receive any support/ instruction? If not, where did they get the knowledge on how to prepare them?

3c. Current situation

- How do they feel now? Have their feelings/attitudes changed over time?
- What are they doing now? Possible topics: participation in training? teacher cooperation? whole-school approach?
- Are they offered any support, e.g. administrative staff support/human relief agency involvement/local authority/national/ministerial level?
- Are cultural assistants employed?
- Are language preparation classes offered for children with migration experience? In what format? Where (school, library)?
- Are they involved in any form of training/professional development?
- What action (if any) did they take?

3d. Integration of pupils with migration background

- How were they welcomed by their peers?
- What was it like at the beginning? A period of silence/no-interaction?
- To what extent are pupils with migration background integrated into the school environment now?

3d. Cooperation with parents

- Has there been cooperation with parents? If so, in what ways/areas?
- Did parents take any initiatives themselves?
- Are there differences in the form and extent of parental involvement of Polish parents and parents of children with migration background?
- Plans for parental involvement in the future - any initiatives on the part of the school/parents?
- Problematic areas and how to deal with them
- Additional comments/reflections

4. Session wrap-up

Thank the respondent and provide contact details for sharing findings of the study
